# Competitive Recovery–Stress and Mood States in Mexican Youth Athletes

**DOI:** 10.3389/fpsyg.2020.627828

**Published:** 2021-01-12

**Authors:** Luis Felipe Reynoso-Sánchez, Germán Pérez-Verduzco, Miguel Ángel Celestino-Sánchez, Jeanette M. López-Walle, Jorge Zamarripa, Blanca Rocío Rangel-Colmenero, Hussein Muñoz-Helú, Germán Hernández-Cruz

**Affiliations:** ^1^Departamento de Ciencias Sociales y Humanidades, Universidad Autónoma de Occidente, Los Mochis, Mexico; ^2^Departamento de Investigación, Iniciativa Juvenil Colimense A.C., Colima, Mexico; ^3^Centro de Investigación de Estadística Multivariante Aplicada, Universidad de Colima, Villa de Álvarez, Mexico; ^4^Facultad de Organización Deportiva, Universidad Autónoma de Nuevo León, San Nicolás de los Garza, Mexico; ^5^Departamento de Ciencias Económico-Administrativas, Universidad Autónoma de Occidente, Los Mochis, Mexico

**Keywords:** RESTQ-sport, POMS (profile of mood states), gender differences, individual vs. team sports, confirmatory factor analysis-CFA, Mexico

## Abstract

**Background:**

Monitoring recovery–stress balance in sport is becoming more relevant to prevent training maladaptation and reach the optimal performance for each athlete. The use of questionnaires that identify the athlete’s recovery–stress state have much acceptance in sports due to reliability and useful, furthermore for its low cost. Identifying possible differences between sport modalities and sex is important to determine specific needs and possible intervention ways to keep a recovery–stress balance. The aim was to analyze the differences in the recovery–stress state and mood states by sex and sport type during the competitive phase in young Mexican athletes. As a secondary objective, the psychometric properties of the Mexican version of the Recovery–Stress Questionnaire for Athletes (RESTQ-Sport) were analyzed.

**Methods:**

A cross-sectional study was carried on with 461 athletes (61% women and 39% men), 17.95 (±1.2) years old, from six sports disciplines. The RESTQ-Sport and Profile of Mood States (POMS) were applied in a single moment. Differences by sex and sports modality were analyzed. RESTQ-Sport’s confirmatory factor analysis was performed after the stress and recovery theoretical structure of two stress (general and sport) and two recovery (general and sport) dimensions, and last, the concurrent validation with the POMS was carried on.

**Results:**

Significant differences by sex were found in the General Recovery and Sport Stress dimensions of the RESTQ-Sport as well as Vigor factor of the POMS, being higher for men; furthermore, both the Sport Recovery dimension of RESTQ-Sport and Cholera and the Fatigue and Depression factors from POMS also had differences by sport type, showing a less recovery and high stress for individual sport athletes. Goodness-of-fit indexes of the model for the RESTQ-Sport were acceptable. Pearson’s correlation between questionnaires was moderate (*p* < 0.05).

**Conclusion:**

The recovery–stress state shows differences in the function of sex and sport modality. More special attention is suggested for women and individual sport athletes. The higher punctuation for men compared with women in sport stress dimension did not negatively affect the recovery–stress balance for male athletes. Finally, the Mexican context adaptation of the RESTQ-Sport provides a psychometric instrument suitable to assess the recovery–stress balance in Mexican athletes.

## Introduction

High demands of sports competition require an intelligent approach to schedule training loads and recovery periods (Elbe et al., 2016). Elevated training loads are associated with higher levels of physical and psychological stress (Nunes et al., 2014), which, along with factors such as competition, performance self-assessment, poor recovery, and contextual aspects, can lead the athlete to decreased performance due to excessive fatigue, overload, and overtraining (Kellmann, 2010).

Overtraining syndrome is characterized by a lack of energy, eating, and sleep disturbances. Among other symptoms, it affects moods and concentration (Halson, 2014), highlighting recovery as an essential element to achieve optimal athletic performance. Recovery, understood as a multilevel intra- and interindividual process that can have different periods, is a key factor for sports training (Kellmann et al., 2018), so coaches and athletes should focus on keeping and regaining the body’s balance between the imposed demands and the available physical and psychosocial resources, allowing athletes to train more and improve their skills (Kellmann, 2010). Therefore, attention to recovery processes from a preventive approach involves the increased control of training load impact and avoid athlete’s non-functional maladaptation, as well as promote effective coping strategies to manage stress levels (Heidari et al., 2019; Lope and Solís, 2020).

There are different methods for stress evaluation during training and competition, being the self-report and daily questionnaires reliable tools widely used, that offer non-invasive and low-cost immediate information of the athlete’s state (Kellmann, 2010; Halson, 2014; Nässi et al., 2017). The Profile of Mood States (POMS, McNair et al., 1971), for example, makes it possible to identify overtraining profiles from athletes, evaluating mood changes, which are reflected through their negative and positive factors.

Related studies regarding the impact of high competition on athletes’ moods suggest that factors such as age and competition level can influence the modulation of these states, being better in older and more experienced athletes (Peñaloza et al., 2016). On the other hand, there is a consensus that maintaining a positive profile (iceberg profile), where vigor is above the other factors, can predict optimal sports performance, whereas an inverted iceberg profile (vigor below the negative factors) may be associated with the overtraining syndrome and inadequate stress coping by the athlete, in addition to poor performance (Vidal Andreato et al., 2014; Correia et al., 2016).

In the same way, the Recovery–Stress Questionnaire for Athletes (RESTQ-Sport) arises as a response to the need for a more comprehensive and precise evaluation for psychological symptoms associated with the overtraining syndrome, as well as the athlete’s coping tools to face the situation (Kellmann and Kallus, 2001, 2016). Its capability to evaluate not only the stress levels and sources but also the recovery process in a multidimensional way has made it one of the most applied instruments in stress–recovery monitoring (Kellmann, 2010).

Previous research (Nederhof et al., 2008; González-Boto et al., 2009; Molinero et al., 2012) has identified positive correlations between RESTQ-Sport scales of stress and POMS negative factors, as well as between RESTQ-Sport recovery scales and the vigor factor of the POMS, otherwise (negative correlations) between the stress scales and the vigor factor and the recovery scales of the RESTQ-Sport with the POMS negative factors. Authors conclude that there is a concurrent validity between the questionnaires due to the relationship between parameters associated with overtraining factors (stress and negative moods) and the positive factors and scales of the instruments (vigor and recovery).

Other studies focused on analyzing the relationship between precompetitive anxiety levels and athletic performance; for example, one of them (León-Prados et al., 2014) identified a relationship between cognitive anxiety levels that influenced athletic performance in basketball players. However, Reynoso-Sánchez et al., 2017 identified changes in recovery–stress balance, heightening stress, and decreasing recovery levels at the end of a handball competition regarding precompetition evaluations. In the same way, Vacher et al. (2017) report the influence of the recovery–stress states over the emotional responses in swimmers and the relationship with external and perceived training loads.

On the other hand, it has been previously reported that the sport type (Valcarce, 2011; Garinger et al., 2018) and sex (Holden et al., 2019) have a modulating effect on the perception of stress; similar to other psychological variables such as depression (Nixdorf et al., 2016), both being significantly higher for athletes who practice individual sports. However, more research centered on these differences is needed to be able to get an intervention process that promotes the monitoring and maintaining recovery stress balance with more precision in the future.

Therefore, the present study aimed to analyze the differences by sport type and sex of the recovery–stress levels and mood states of young Mexican athletes during a competitive period. As a secondary objective, we intend to confirm the factorial structure of the four dimensions for the Mexican adaptation of the RESTQ-Sport and examine the internal consistency and concurrent validity with the POMS factors in young Mexican athletes. Researchers hypothesize that the evaluated athletes will have differences in sex and sport-type practice, with perceived stress and negative moods being greater for both women and athletes of individual sports.

## Materials and Methods

### Participants

A cross-sectional study was carried out with explanatory-correlational scope. A convenience non-probabilistic sampling was used. Four hundred sixty-one athletes who participated in the National Youth Championship in 2016 (61% women and 39% men), age 17.95 (±1.2 years; range = 16–20) years old, participating at six different sports disciplines (athletics = 10.4%; soccer = 15.2%; handball = 31.7%; racquetball = 2.8%, taekwondo = 21%, water polo = 18.9%) from 26 of the 32 states of the Mexican Republic took part in this study.

### Instruments

#### Recovery–Stress Questionnaire for Athletes 76

Recovery–stress balance was assessed with the RESTQ-Sport 76 (Kellmann and Kallus, 2001, 2016). Athletes indicated how often they participate in different activities and experience different feelings and emotions during the previous 3 days and nights. They answer a seven-point Likert-type scale (0 = never to 6 = always). This questionnaire consists of 76 items distributed in 19 scales that are grouped in four dimensions (Kellmann and Kallus, 2016): There are seven general stress (GS), five general recovery scales (GR), three sport stress (SS), and four Sport Recovery scales (SR). The original proposal of Kellmann and Kallus (2001, 2016) indicates two questionnaire dimensions: stress and recovery, gathered in a general and sports factor for each dimension, which determines the Recovery–stress balance (RSB) by subtracting the total recovery from the total stress. A positive RSB means an adequate balance.

The instrument measures the relationship between activities, experienced moods, and events with respect to current stress and recovery, identifying patterns based on perceived physical and psychosocial stress and the athlete’s coping resources, explaining the factors that can generate overtraining in athletes (Kellmann and Kallus, 2016). A positive recovery–stress balance (higher levels of recovery perceived overstress) is associated with athletes’ ability to cope with the demands, whereas an imbalance indicates coping inability and overload produced by training and context.

Recovery–Stress Questionnaire for Athletes has been validated in different languages: English (Kellmann and Kallus, 2001, 2016), Spanish (González-Boto et al., 2008), Dutch (Nederhof et al., 2008), and French (Martinent et al., 2014). Additionally, it has been used in different sports such as soccer (Laux et al., 2015), basketball (Nunes et al., 2014), volleyball (Freitas et al., 2014), cycling (Filho et al., 2013), and swimming (Elbe et al., 2016; Vacher et al., 2017), among others. The factorial proposal of the instrument was corroborated by González-Boto et al. (2008) and Nicolas et al. (2019). The internal consistency coefficient of the entire questionnaire and each factor has been higher than 0.70 (González-Boto et al., 2008; Nederhof et al., 2008; Martinent et al., 2014), which allows the acceptance of the model.

The use of the RESTQ-Sport has been reported in four studies with Mexican samples; three of them using a Mexican context adapted version. All of them had adequate Cronbach’s α values (>0.70). The first was carried out with volleyball players (20.75 ± 1.94 years old), reporting α-values greater than 0.80 on the global scale and 0.90 for each dimension (Reynoso-Sánchez et al., 2016). The second assessed pre-, during, and post-competition in handball players (22.3 ± 1.8 years), with internal consistency coefficients greater than 0.85 for each time (Reynoso-Sánchez et al., 2017). The third was realized with endurance runners (20.1 ± 2.7 years), indicating values of α above 0.70 (Hernández-Cruz et al., 2017). The fourth study was performed with soccer players using the Spanish version of RESTQ-Sport, obtaining a Cronbach’s α > 0.80 (Lope and Solís, 2020). However, no other psychometric properties analyzing the validity of RESTQ-Sport have been reported.

#### Profile of Mood States

To assess mood states, the POMS scale was applied (McNair et al., 1971). This instrument allows a multidimensional evaluation of mood states throughout adjectives related to different factors (Nässi et al., 2017). Participants answered a retrospective questionnaire of the last 3 days/nights from a five-point Likert scale (0 = nothing and 4 = very much). Originally, the POMS was structured by seven scales, but according to the analysis from Arce et al. (2000), the six-scales version has better reliability and validity indices for the application with athletes. Following this purpose, the 58 items grouped in the six-factor version were applied; five negatives: (a) tension, (b) depression, (c) anger, (d) fatigue, and (e) confusion; and one positive: (f) vigor (Arce et al., 2000).

### Procedure

The study procedure was conducted by two main phases: the first one to adapt the RESTQ-Sport to the contextual characteristics of the Spanish language in Mexico, and the second phase consisted of the process for the questionnaires’ application during the National Championship. For the RESTQ-Sport adaptation to the Mexican context phase, the method proposed by Muñiz et al. (2013) was followed. Two translators with experience in adapting psychometric tests performed the English to Spanish translation and back to English. Subsequently, an English native language translator carried out the qualitative evaluation between questionnaires, the original and the translated versions (English–Spanish–English). Finally, a pilot test with 10 athletes was performed to assess the understanding of the questionnaire, obtaining a satisfactory evaluation.

Finally, to achieve the application phase, first, the authorization to apply both questionnaires (RESTQ-Sport and POMS) was requested to the organization of the National Youth Championship. Once permission was obtained, a team of 10 volunteers was recruited and trained for the correct application of the questionnaires. The application team was composed of psychology and physical activity and sport students. One day before the start of the application, the researchers and team had a meeting to explain the study’s purpose and the specifications for application instructions of the questionnaires. Each volunteer answered the questionnaire as a practical example to identify possible doubts and learn the correct way to solve them for athletes if necessary. The support from the directors of each delegation was solicited, and the participants’ consent was requested. The researchers scheduled the date for the application of the questionnaires with the directors of each delegation before the competition start. The questionnaire was applied at the best possible relaxed and calm conditions at the hotel lobby where the athletes were staying. Volunteers and researchers supervised during the application of the questionnaires, asking athletes to respond to all items and answering their doubts. The entire application process followed the agreements of the Declaration of Helsinki (World Medical Association, 2013) to ensure an ethical approach with the participants and the treatment of the collected data.

### Statistical Analyses

Descriptive analysis for means (*M*), standard deviation (*SD*), skewness, and kurtosis was performed for the RESTQ-Sport and the POMS. A ± 2 for asymmetry and kurtosis was considered as acceptable normality ranges (Kim, 2013). The RESTQ-Sport scales were grouped into their respective dimensions and the POMS factors for the analysis of the differences in the perception of stress–recovery and mood states. Subsequently, a Student *t*-test for independent samples was performed, assuming the equality of the variance by sex (woman and man) and sport type (individual or team) as independent factors. Values of *p* < 0.05 were considered as significant differences.

Based on the RESTQ-Sport theoretical postulate, we proceeded to confirmatory factor analysis, grouping the scales in each of the four corresponding theoretical dimensions. Due to the high number of items of the RESTQ-Sport in relation to the sample, according to the recommendations of Ruíz et al. (2010), the analysis by the scales had to be carried out. The maximum likelihood method was chosen for continuous variables of the normal distribution as an estimation procedure. The following goodness-of-fit statistics were considered for the validity of the instrument: relative chi-square (*x*^2^/df), values below five are considered acceptable (Wheaton et al., 1977); the square root of the approximation error and the standardized square root of the residual, which must be equal to or below 0.08 to be considered appropriate (Ruíz et al., 2010); the comparative fit index and the goodness-of-fit index, appropriate parameters when they are greater than or equal to 0.90 (Byrne, 2010); and the corrected goodness-of-fit index considering adequate values greater than 0.80 (Pérez-Gil et al., 2000).

The internal consistency for each dimension of the instrument was evaluated using Cronbach’s alpha (Cronbach, 1951), McDonald’s omega (McDonald, 1999) based on confirmatory factor analysis, and the average variance extracted (AVE). For Cronbach’s alpha and McDonald’s omega, values greater than 0.70 were considered acceptable (Hair et al., 2009). For the AVE, it is suggested that its value exceed 0.50 (Hair et al., 2009). To evaluate concurrent validity, a Pearson correlation analysis was performed between the RESTQ-Sport and the POMS scales. For the data analysis, the statistical packages SPSS and AMOS v. 22 were used.

## Results

### Assessment Based on Sex and Sport Type

Table 1 shows the *M* ± *SD* of the RESTQ-Sport dimensions and the POMS factors. Regarding the analysis for recovery–stress level differences by sex, men show significantly higher levels in the GR [*F*(459) = 3.56; *p* = 0.000], SS [*F*(459) = 0.48; *p* = 0.041], and SR [*F*(459) = 7.11; *p* = 0.006] dimensions of the RESTQ-Sport, as well as a greater perception in vigor factor [*F*(459) = 2.68; *p* = 0.000] of the POMS. Although the analysis of variance by sport type revealed that individual sport athletes present according to RESTQ-Sport, more SS [*F*(459) = 0.029; *p* = 0.014] and less GR [*F*(459) = 7.79; *p* = 0.034], as well as a greater negative mood states perception, being higher for anger [*F*(459) = 8.45; *p* = 0.047], fatigue [*F*(459) = 17.63; *p* = 0.000], and depression [*F*(459) = 15.26; *p* = 0.007] POMS factors, opposed to the vigor factor [*F*(459) = 2.21; *p* = 0.050] in which the team athletes show a higher levels.

**TABLE 1:** Means ± SD and variance analysis of RESTQ-Sport and POMS.

	Sex			Sport type		
	Women	Men		Individual	Team	

	*M* ± SD	*M* ± SD	*t*	*M* ± SD	*M* ± SD	*t*
GS	2.00 ± 0.88	1.98 ± 0.86	0.07	1.96 ± 0.88	2.01 ± 0.87	0.09
GR	3.87 ± 0.76	4.16 ± 0.70	3.56**	3.88 ± 0.85	4.05 ± 0.69	7.79*
SS	2.22 ± 1.10	2.43 ± 1.05	0.48*	2.48 ± 1.09	2.21 ± 1.07	0.03**
SR	4.00 ± 1.01	4.24 ± 0.88	7.11**	4.06 ± 1.02	4.11 ± 0.94	0.84
RSB	1.83 ± 1.38	2.00 ± 1.33	1.30	1.75 ± 1.48	1.96 ± 1.29	8.04
Tension	1.41 ± 0.73	1.38 ± 0.63	4.75	1.37 ± 0.74	1.41 ± 0.67	2.58
Depression	0.64 ± 0.65	0.60 ± 0.63	0.01	0.74 ± 0.74	0.56 ± 0.57	15.26**
Anger	0.92 ± 0.75	0.93 ± 0.66	4.34	1.02 ± 0.80	0.88 ± 0.67	8.45*
Vigor	2.26 ± 0.66	2.56 ± 0.56	2.67**	2.29 ± 0.67	2.42 ± 0.62	2.21*
Fatigue	0.92 ± 0.77	0.89 ± 0.72	1.34	1.12 ± 0.85	0.80 ± 0.67	17.63**
Confusion	1.00 ± 0.66	1.00 ± 0.63	0.27	1.06 ± 0.69	0.97 ± 0.62	2.11

GS, general stress; GR, general recovery; SS, sport stress; SR, sport recovery; RSB, recovery–stress balance. *p < 0.05; **p < 0.01.

### Psychometric Properties of Recovery–Stress Questionnaire for Athletes in Mexican Context

The univariate statistics for the instrument dimensions indicated a normal distribution, as the asymmetry ranges were from −0.84 to 0.66 and the kurtosis ranges from −0.57 to 0.60. GS dimension was the one that presented the highest internal consistency (α = 0.89; *ω* = 0.90; AVE = 0.56), followed by SR (α = 0.87; *ω* = 0.88; AVE = 0.65), SS (α = 0.78; *ω* = 0.82; AVE = 0.60), and GR (α = 0.73; *ω* = 0.75; AVE = 0.38). The internal consistency of the entire instrument (α = 0.74) was higher than the minimum acceptable parameters.

**FIGURE 1:**
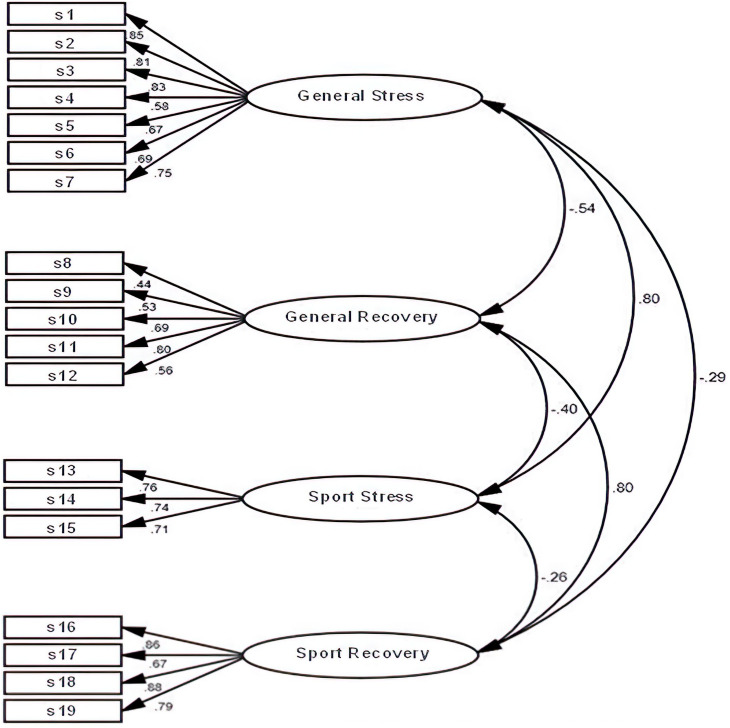
Four-factors model of the RESTQ-Sport in a young Mexican athletes’ sample [*N* = 461, (x2/df) = 4.79, RMSEA = 0.09, SRMR = 0.09, CFI = 0.90, GFI = 0.90, AGFI = 0.80]. Confirmatory factor analysis represents the general stress, general recovery, sport stress, and sport recovery dimensions in ovals (latent variables). Nineteen scales (observed variables), which comprise 76 items, are reflected in rectangles. Values showed above arrows indicate the standardized regression weights. s1, general stress; s2, emotional stress; s3, social stress; s4, conflicts/pressure; s5, fatigue; s6, lack of energy; s7, physical complaints; s8, success; s9, social recovery; s10, physical recovery; s11, general well-being; s12, sleep quality; s13, disturbed breaks; s14, emotional exhaustion; s15, injury; s16, being in shape; s17, personal accomplishment; s18, self-efficacy; s19, self-regulation.

Through the confirmatory factor analysis, it was found that stress factors (GS and SS) and recovery factors (GR and SR) were positively correlated with each other, both with a coefficient of 0.80 (Figure 1). In the opposite way, GS and SS factors correlated negatively with GR and SR factors, which was in line with what was theoretically expected. The factorial loads for the scales in each dimension were found above the value 0.40 (Pérez and Medrano, 2010).

When reviewing the goodness-of-fit indexes of the model, it is possible to observe that, in general, they reflected a satisfactory fit, being the (*x*^2^/df) = 4.79, the root mean square error of approximation (0.09), standardized root mean residual (0.09), whereas the comparative fit index (0.90) and goodness-of-fit index (0.90) and adjusted goodness-of-fit index (0.80) are within the accepted ranges for the model fit.

### Correlations Between Questionnaires

Regarding the POMS scales analysis, the questionnaire showed acceptable univariate normality ranges (−0.36 to 1.37 of asymmetry and −0.09 to 1.34 of kurtosis) and values total internal consistency (α = 0.82). Finally, correlations between the scales of the questionnaires (Table 2) were observed. The five negative POMS factors correlated (*p* < 0.05) positively with the stress scales and negatively with the recovery scales of the RESTQ-Sport. In the opposite way, the positive factor (vigor) of the POMS presented positive associations (*p* < 0.05) with the recovery scales of the RESTQ-Sport; however, only three stress scales correlated negatively with vigor and one of them (conflicts/pressure) positive. According to our results, it is possible to point out that the Mexican version of RESTQ-Sport shows concurrent validity with the POMS.

**TABLE 2:** Pearson correlations between RESTQ-Sport Scales and POMS factors.

RESTQ-Sport	POMS					
	Tension	Depression	Anger	Vigor	Fatigue	Confusion
General stress	0.454**	0.569**	0.561**	−0.113*	0.504**	0.548**
Emotional stress	0.462**	0.439**	0.523**	–0.035	0.408**	0.431**
Social stress	0.418**	0.458**	0.531**	–0.073	0.428**	0.442**
Conflicts/Pressure	0.424**	0.311**	0.299**	0.102*	0.352**	0.320**
Fatigue	0.344**	0.371**	0.367**	–0.003	0.546**	0.338**
Lack of energy	0.427**	0.408**	0.354**	–0.034	0.398**	0.474**
Physical complaints	0.433**	0.409**	0.389**	–0.042	0.490**	0.412**
Success	−0.138**	−0.210**	−0.130**	0.313**	−0.134**	−0.257**
Social recovery	–0.055	−0.175**	−0.107*	0.318**	−0.164**	−0.176**
Physical recovery	−0.272**	−0.296**	−0.206**	0.424**	−0.313**	−0.344**
General well-being	−0.293**	−0.395**	−0.352**	0.432**	−0.376**	−0.375**
Sleep quality	−0.351**	−0.328**	−0.295**	0.163**	−0.376**	−0.359**
Disturbed breaks	0.337**	0.352**	0.382**	–0.050	0.472**	0.395**
Emotional exhaustion	0.376**	0.497**	0.456**	−0.137**	0.541**	0.473**
Injury	0.372**	0.325**	0.354**	0.009	0.493**	0.330**
Being in shape	−0.267**	−0.273**	−0.184**	0.468**	−0.324**	−0.364**
Personal accomplishment	−0.186**	−0.213**	−0.155**	0.323**	−0.194**	−0.254**
Self-efficacy	−0.269**	−0.291**	−0.141**	0.454**	−0.261**	−0.345**
Self-regulation	−0.186**	−0.190**	−0.097*	0.396**	−0.207**	−0.288**

*p < 0.05; **p < 0.01.

## Discussion

The purpose of this study was to analyze the differences in the perceived recovery–stress state and the moods states based on sex and sports type on young Mexican athletes before a competition. According to the review carried out by the authors, this is the first study in Mexico with these characteristics, being one of the few that analyzes the differences in the behavior of the questionnaire by sex, in addition to being one of the studies with the largest sample that applies the RESTQ-Sport.

Based on sex results, it was possible to observe that women present lower levels of perceived GR and SR, as well as lower vigor compared with men. This coincides with what has already been reported in research that contrasted the stress–recovery behavior between female and male basketball players (Di Fronso et al., 2013) and rowers (Kellmann et al., 2001), as well as with the results referent to mood states reported by Brandt et al. (2017), which confirms the need for individualization for both, the loads proposed, and monitoring the internal training load on athletes, demanding more precise attention for female athletes to prevent poor training adaptations and overtraining syndrome.

Continuing with the comparisons by sex, a novel finding of the present investigation is that men report levels of SS significantly higher (*p* < 0.05) than women, thus contrasting with other reports where women tended to present more stress (Kellmann et al., 2001; Di Fronso et al., 2013; Holden et al., 2019). Notwithstanding, the RSB was higher for men, which may be due to the coping and strategies skills used by them to counteract the stress demands (Molinero et al., 2012).

Regarding the appraisal by sport type, our results coincide with what was reported in previous research (Nixdorf et al., 2016; Brandt et al., 2017; Garinger et al., 2018). Athletes who practice individual sports have higher SS levels and lower GR (*p* < 0.05) and the presence of higher negative moods such as depression, anger, and fatigue, whereas vigor is lower. Some authors refer that the requirement for individual sports is reflected on the stress levels perceived by their athletes (Garinger et al., 2018); instead, the support of teammates and together problem solving allow those who participate in team sports to apperceive better recovery levels, and they are less likely to present symptoms related to excessive levels of stress and even depression (Nixdorf et al., 2016).

As a secondary research objective, an analysis of the properties of validity for adaptation of the RESTQ-Sport to the Mexican population was proposed. The analysis indicates that the instrument has adequate psychometric properties to be a valid measure. The Cronbach α values indicate that the RESTQ-Sport is reliable both globally and divided into its four dimensions (GS, GR, SS, and SR), which coincides with the indexes reported in other languages being above 0.60 (Kellmann and Kallus, 2001, 2016; González-Boto et al., 2008; Nederhof et al., 2008), as well as was previously indicated in samples with Mexican athletes that obtained α-values greater than 0.70 (Reynoso-Sánchez et al., 2016, 2017; Hernández-Cruz et al., 2017). On the other hand, this is because of the general scales from the RESTQ for the general population, which were taken as a basis for the RESTQ-Sports; likewise, diversity of the disciplines from the sample in our study constitutes factors that could affect the reliability coefficient, which has already been reported in previous research (González-Boto et al., 2008; Kellmann and Kallus, 2016).

Related to the model structure, results show the multidimensional nature for the constructs evaluated, both the dimensions associated with stress and the others to recovery. This two-dimensionality has been empirically corroborated in several studies making the theoretical perspective adopted by Kellmann and Kallus increasingly robust (Kellmann and Kallus, 2001, 2016). Following the recommendations proposed by Bazán et al. (2006), the study was based on the previous empirical evidence and the researcher’s criteria to contrast the structural model with four factors through confirmatory factor analysis.

The analysis of structural models was carried out following the authors’ guidelines of the questionnaire. Four specific factors were grouped, two for stress and two for recovery. Correlations between GS and SS factors were positive and high, as well as between GR and SR, agreeing with what was reported by González-Boto et al. (2008). It was also observed that six goodness-of-fit indices of the confirmatory factor analysis showed an adequate fit. Although the parameter (*x*^2^/df) received several criticisms due to its high sensitivity to sample sizes and is based on the central distribution of *x*^2^ (Byrne, 2010), it is convenient finding a model more accurately representing the underlying structure of the data. Therefore, future research should contrast these results and analyze in greater depth the psychometric characteristics of the questionnaire in the Mexican context. Perspectives, such as the item theory response or the analysis of the impact of the items, represent valuable approaches that could contribute both to the general study of the instrument and for each scale and its items.

The last objective of the study was to prove the concurrent validity of the RESTQ-Sport by comparing it with the POMS. Data show a positive correlation (*p* < 0.05) between the RESTQ-Sport stress scales with POMS factors such as tension, depression, anger, fatigue, and confusion, whereas recovery scales did negatively with them, thus fulfilling what was theoretically expected (Kellmann and Kallus, 2001, 2016). The result coincides with the analysis reported by Nederhof et al. (2008) and González-Boto et al. (2009), who have applied this method to provide greater robustness to the constructs through the comparison between the instruments in another populations and languages, as well as with the results of Molinero et al. (2012).

### Limitations and Future Research Directions

The study has several limitations; the principal was the ages and sport level of the participants, which was limited only to youths who competed at the national championship. Due to the national championship nature, the authors limited the sample; so, future research must be focused on older samples and more experienced sport level to analyze the recovery–stress behavior of Mexican athletes. Another research limitation is the cross-sectional collection of the data; participants were in a competitive situation, this for sure affected their moods and recovery–stress perception, showing only a situational state that could be altered by not controlled variables such a long travel to the event place, recent injuries, among other factors. More comparisons between sex and sport modalities should be carried on through longitudinal studies to enhance the knowledge over the recovery–stress states and their fluctuations during specific training or session periods in relation to sex or type of sports practice.

On the other hand, the goodness-of-fit indexes of the model for the RESTQ-Sport are on the limit for an adequate fit; this could be because of the size of the sample, being theoretically the minimum number of subjects for an item of the questionnaire. It is recommended for future research in Mexican athletes that the reliability analysis will carry on every application of the RESTQ-Sport to give more robustness to the instrument.

### Conclusion

According to the research results, it is possible to conclude that in the athletes of the present study, there are differences in the perception of recovery–stress state between men and women, demonstrating the need to focus on promoting more effective coping strategies for young female Mexican athletes that enable them to enhance the perception of recovery, both in general and at sports level. In the same way, implement training loads and recovery–stress balance monitoring to prevent negative psychological and physical symptoms caused by overtraining syndrome or burnout. This assessment must be more precise for athletes who practice individual sports and for female Mexican athletes. Finally, the RESTQ-Sport questionnaire adaptation to the Mexican context proved to be a valid and reliable instrument and efficient and accurate for monitoring the recovery–stress state in young Mexican athletes, being a useful and economic tool to prevent and/or detect overtraining.

## Data Availability Statement

The raw data supporting the conclusions of this article will be made available by the authors, without undue reservation.

## Ethics Statement

Ethical review and approval was not required for the study on human participants in accordance with the Local Legislation and Institutional Requirements. Written informed consent to participate in this study was provided by the participants’ legal guardian/next of kin.

## Author Contributions

LR-S, JL-W, JZ, and GH-C: design of the work. LR-S and GH-C: investigation and project application. LR-S, GP-V, MC-S, JL-W, and JZ: formal analysis and interpretation of the data for the work. LR-S, GP-V, and GH-C: writing–original draft preparation. JL-W, JZ, BR-C, and HM-H: writing–review and editing. JL-W, BR-C, and HM-H: funding acquisition. All authors have read and agreed to the published version of the manuscript.

## Conflict of Interest

The authors declare that the research was conducted in the absence of any commercial or financial relationships that could be construed as a potential conflict of interest.
